# Using informative features in machine learning based method for COVID-19 drug repurposing

**DOI:** 10.1186/s13321-021-00553-9

**Published:** 2021-09-20

**Authors:** Rosa Aghdam, Mahnaz Habibi, Golnaz Taheri

**Affiliations:** 1 School of Biological Sciences, Institute for Research in Fundamental Sciences (IPM), Tehran, Iran; 2 Department of Mathematics, Qazvin Branch, Islamic Azad University, Qazvin, Iran; 3 Department of Electrical Engineering and Computer Science, KTH Royal Institute of Technology, Stockholm, Sweden; 4 Science for Life Laboratory, Stockholm, Sweden

**Keywords:** Coronavirus disease 2019, SARS-CoV-2, Protein−protein interaction, Clustering method

## Abstract

Coronavirus disease 2019 (COVID-19) is caused by a novel virus named Severe Acute Respiratory Syndrome Coronavirus-2 (SARS-CoV-2). This virus induced a large number of deaths and millions of confirmed cases worldwide, creating a serious danger to public health. However, there are no specific therapies or drugs available for COVID-19 treatment. While new drug discovery is a long process, repurposing available drugs for COVID-19 can help recognize treatments with known clinical profiles. Computational drug repurposing methods can reduce the cost, time, and risk of drug toxicity. In this work, we build a graph as a COVID-19 related biological network. This network is related to virus targets or their associated biological processes. We select essential proteins in the constructed biological network that lead to a major disruption in the network. Our method from these essential proteins chooses 93 proteins related to COVID-19 pathology. Then, we propose multiple informative features based on drug–target and protein−protein interaction information. Through these informative features, we find five appropriate clusters of drugs that contain some candidates as potential COVID-19 treatments. To evaluate our results, we provide statistical and clinical evidence for our candidate drugs. From our proposed candidate drugs, 80% of them were studied in other studies and clinical trials.

## Introduction

The pandemic situation for Coronavirus disease 2019 (COVID-19) causes more than 197 million infections and more than 4.2 million deaths in more than 200 countries worldwide (until the end of July 2021) and this number is increasing rapidly. Due to this rapid spread, researchers have been searching for therapeutic approaches in the past few months. At present, no medicine has been claimed to be effective in the treatment or even prevention of COIVD-19 [1]. On the other hand, producing new drugs with a complete drug profile is a tough task that requires extensive time and budget. Drug repurposing is the procedure of perusing new therapeutic uses for available drugs. This process can reduce a large amount of time, money, and danger of the traditional drug discovery process [2]. The main purpose of drug repurposing is to exceed the therapeutic use of the available drugs for more medical scope. Previous researches showed that drugs with similar profiles probably demonstrate similar behavior in the existence of similar targets like proteins [1–3]. Traditional drug repurposing methods are mainly based on finding the relationship between biological activity and the molecular structure of different drugs. However, newer data gathering and analysis shows the urgent need for using computational methods for drug design and repurposing. Computational methods are mainly used to discover different drug interactions that are not considered and found during the clinical trial process [4]. In drug repositioning, computational methods investigate the relationship between drug databases and genomic, transcriptomic, and other available information with the help of data and network analysis and machine learning methods [2]. Machine-learning based methods for drug repurposing reveal the connection between drugs, viral, and host proteins. In the life cycle of a virus, the viral proteins are associated with different human proteins in the infected cells through different interactions. Within these interactions, the virus hijacks the host cells for replication, and this process changes the regular function of these interacted proteins. Therefore, to design antiviral drugs, a complete understanding of the interaction between human proteins and viral is crucial [5]. It is worth mentioning, in drug repurposing to fight the virus, targeting just virus proteins is not the proper approach. Targeting single virus proteins can cause the viruses to escape this attack through some backup pathways. These backup pathways lead to increased virus resistance with the mutation. Host-directed treatments propose significant strategies [6]. These methods select human proteins as the main carriers for the virus to enter and control human cells. These host-directed treatments seem to be less susceptible to making resistance because human proteins are less influenced by mutations. Therefore, targeting human proteins as drug targets is a more sustainable strategy. In host-directed treatments, it is important to find proteins that are essential for the maintenance and persistence of the disease that is caused by a virus in the human cells. When these proteins are targeted as drug targets, the replication mechanism of the virus collapses. For all of the above-mentioned reasons, repurposing drugs with host-directed treatments against COVID-19 has major potential. Furthermore, drug repurposing methods provide hope for fast practical implementation with the minimum side effects. Molecular interaction and biological interaction networks as valuable resources are the foundation for drug repurposing methods [7]. This means that network-based drug repurposing methods propose novel opportunities for finding drug targets in host-directed treatments [8]. Recent studies show that valuable results are based on viral-host networks for treating HIV [9], Hepatitis C [10], and Ebola as well [11]. Since the outbreak of COVID-19 some research groups have been trying to develop network-based methods to find some repurposed drugs to operate against SARS-CoV-2. Zhou et al. [12] proposed a network-based method for the identification of some candidates as repurposable drugs and some potential drug combinations targeting. Li et al. [13] combined network data with a relative analysis of the gene sequences of the different viruses to find potential drugs for SARS-CoV-2. Gordon et al. [14] proposed a map from human proteins with SARS-CoV-2 proteins that were found to interact in the affinity purification mass spectrum method. Dick et al. [15] recognized high confidence interactions between human proteins and SARS-CoV-2 proteins with the help of sequence-based protein−protein interaction (PPI) predictors.

In this paper, we propose the four steps method. This method tries to identify novel drug targets and pathways associated with essential proteins in COVID-19. In the first step, we build a graph as a COVID-19 related biological network related to virus targets or their associated biological processes. In the second step, we use two effective algorithms [16, 17] to find the candidate set of proteins from biological networks that lead to a major disruption in the network. In the third step, we identify proteins in our candidate set that are associated with some underlying diseases related to COVID-19. Then, we select 93 proteins as a final set of essential proteins related to disease pathology. Identifying essential proteins may elucidate new drug targets and pathways related to COVID-19. In the fourth and last step, we propose informative features based on drug-protein and PPI networks and find five significant clusters that contain appropriate candidate drugs. Our results show that using our four steps method suggests some appropriate candidate drugs. Most of these candidate drugs are recommended in other studies.

## Methods

### Finding essential proteins related to COVID-19 pathology as candidate drug targets

Introducing the essential proteins related to COVID-19 pathology as candidate drug targets is one of the most used and appropriate ways to find suitable drugs for COVID-19 treatment. In this subsection, we describe the first, second and third steps of our proposed method. These two steps try to find the set of essential proteins related to COVID-19 pathology. In the first step, we use two effective algorithms [16, 17] for finding the minimum number of proteins that participate in a large number of biological processes. We use these algorithms to find sets of essential proteins based on the disruption of the COVID-19 related biological network. In the second step, we investigated COVID-19 associated protein sets. As a result of this step, we found a subset of essential proteins that are essential to disease pathology.

#### Construction of COVID-19 related biological network

Suppose that informative biological processes (IBP) is a set of biological processes related to virus targets in COVID-19 that will be described in the next subsection. Two proteins are functionally interacted if they are connected through the same biological processes. A COVID-19 related biological network is considered as a weighted undirected graph $G= (V, E, \omega )$. In this graph, each node $v_i \in V$ represents the protein and each edge $e_{ij} \in E$ represents a functional interaction between two nodes $v_i$ and $v_j$. The $\omega (e_{ij} )$ shows the weight of $e_{ij}$ that demonstrates the number of biological processes that two nodes $v_i$ and $v_j$ participate in them. A path between two nodes $v_j$ and $v_k$ in the graph is a sequence of edges that connect the number of distinct nodes through this path. In the weighted graph, the weight of the path between two nodes is defined as follows. Suppose that $v_j$ and $v_k$ as the two ends of this path. Then, the sum of the weight of edges between these two nodes is the weight of this path. A path with the minimum weight between these two nodes is named the shortest path. Now, we define the betweenness value for each node, $v_i$, in the graph in the following way:

1 $$\begin{aligned} Betw ({ v_i}) = \sum _{ { {v_j}, {v_k}} \in {V} } \frac{\theta _{e_{jk}} {v_i} }{\theta _{e_{jk}}}, \end{aligned}$$

where $\theta _{e_{jk}}$ shows the total number of shortest paths from node $v_j$ to node $v_k$ and $\theta _{e_{jk}} {v_i}$ indicates the number of shortest paths that pass through node $v_i$.

#### Disruption of COVID-19 related biological processes

We adapt two algorithms to detect the essential proteins in the COVID-19 related biological network [16, 17]. These algorithms [16, 17] select some of the best candidates as removal proteins set from the COVID-19 related biological network to make a major disruption in it. We place the outputs of Algorithm 1 and 2 in $Cut_1$ and $Cut_2$, respectively.

#### Algorithm 1: spectral partitioning

Partitioning a simple graph, *G*, into disjoint balanced or nearly balanced parts with removing the minimum number of edges between these two parts is known as the *NP*-complete problem [16]. We try to approximate this partitioning problem with the spectral partitioning algorithm. This algorithm is based on eigenvectors of the Laplace of the graph, *G*, and divides the graph into two disjoint parts with respect to eigenvectors of a Laplacian matrix. It is worth mentioning that, the spectral partitioning algorithm is one of the best heuristic approaches for graph partition. Let $A= [a_{ij}]$ shows the adjacency matrix of graph *G* such that,

2 $$\begin{aligned} a_{ij} = {\left\{ \begin{array}{ll} 1 &{} \quad \textit{if } (v_i , v_j) \in { E} \\ 0 &{} \quad \textit{ otherwise} \end{array}\right. } \end{aligned}$$

We define a diagonal degree matrix $D = diag(d_i)$ for graph *G*. In this matrix value $d(v_i)$ shows the degree of $v_i$ in graph *G*. The Laplacian matrix of the graph *G* is defined by $L = D \backslash A$ and $L(G)=[l_{ij}]$ where,

3 $$\begin{aligned} l_{ij} = {\left\{ \begin{array}{ll} 1 &{} \quad \textit{if } (v_i , v_j) \in { E}\\ d(v_{i}) &{} \quad \textit{if } i=j\\ 0 &{} \quad \textit{ otherwise} \end{array}\right. } \end{aligned}$$

The Laplacian matrix is a symmetric positive semi-definite matrix. This matrix has some important properties. Suppose that vector $u=(u_1, u_2, ..., u_n)$ shows the normalized eigenvectors of matrix *L*(*G*) and vector $(\lambda _1, \lambda _2, ..., \lambda _n)$ demonstrates the corresponding eigenvalues of these eigenvectors. We first compute the eigenvectors of Laplacian matrix *L*(*G*), according to the second smallest eigenvalue of this matrix ,$\lambda _2$, and put them in vector $X=(x_1,..., x_n)$. Then, we sort the elements of vector *X* and insert half of the nodes in partition $G_1$ and the reminder of nodes in another partition $G_2$. This procedure divides the nodes of graph *G* into two partitions, $G_1$ and $G_2$ with nearly equal sizes. Removing the edges between these two parts through the cut edges $E(G_1, G_2)$ makes these two parts disconnect. Suppose the vector $A = \{ \alpha _1, ..., \alpha _m \}$ shows the vertices placed in part $G_1$ and vector $B = \{ \beta _1, ..., \beta _m \}$ shows the vertices are placed in part $G_2$, respectively. To make these two parts, $G_1$ and $G_2$ disconnect, we choose vertices from vectors *A* and *B* repeatedly. The vertices are chosen with respect to their degrees and removed until the all edges in $E(G_1, G_2)$ are covered.

#### Algorithm 2: betweenness value

This algorithm [17] tries to make the maximum disruption in the network by removing the minimum number of essential proteins. The selection method in algorithm [17] is based on the betweenness value mentioned in Eq 1. The algorithm [17] has three parts. In the first part, the betweenness value for each node in the graph *G* is calculated. In the second part, to separate the graph *G* into two disjoint partitions $G_1$ and $G_2$, the node with the minimum betweenness value in graph *G* is selected and placed in partition $G_1$. Then, from all of the neighbors of the selected node, the node with the minimum betweenness value is selected and placed in the other partition $G_2$. These procedures are repeated until all nodes are placed into two disjoint partitions $G_1$ and $G_2$. In the third part, the minimum number of nodes from two constructed partitions $G_1$ and $G_2$ is selected with respect to their betweenness values to remove all edges in $E(G_1, G_2)$. The third step of this algorithm is equivalent to the minimum bi-section problem that is an *NP*-complete problem [18].

#### Candidate essential proteins associated with COVID-19 pathology

COVID-19 is a pandemic disease and has different severity and symptoms for various patients. The severity of this disease can vary from asymptomatic to fatal for different people. Recent studies show that this disease has high severity in people with some underlying conditions. Some of the most related underlying diseases are Diabetes, Cardiovascular diseases, Lung diseases, Hepatitis, Kidney disease, and different types of cancer. Hence, we expect that the genetics of these underlying diseases has some correlations with the essential proteins in COVID-19. For finding these essential proteins, we use the relation between gene and disease from Database for Annotation, Visualization, and Integrated Discovery (DAVID). Then, we select some proteins through our two mentioned algorithms that are annotated to four out of five of these specific comorbid diseases. From these selected proteins, proteins with significant p-values as a set of essential proteins associated with COVID-19 are chosen and placed in *E* as a set of main target candidates of COVID-19 drugs.

### Drug clustering method

#### Protein−protein interaction network

We use 5 human high-throughput PPI networks in this work. The first one, Huri, contains 52,248 binary interactions [19]. The second one is collected from the biological general repository for interaction datasets (BioGRID) and contains 296,046 interactions [20]. The BioGRID dataset contains various interactions that are created from different techniques. In this work, we just use the physical interactions between proteins. The three other datasets are human integrated protein−protein interaction reference (HIPPIE) [21], agile protein interactomes dataanalyzer (APID) [22], and homologous interactions (Hint) [23] that contain 57,428, 171,448, and 64,399 experimental interactions, respectively. These interactions are derived from high-throughput yeast-two hybrid (Y2H) and mass spectrometry methods. We map all of the proteins from these five datasets to their corresponding universal protein resource (UniProt) ID [24]. We removed a protein if it could not be mapped to a Uniprot ID. Finally, in this study, we used 25,260 proteins and 304,730 interactions. For all of these proteins, we use biological process terms from gene ontology (GO) term [25] to point out the biological modules in humans. We find that 20,642 proteins from these 25,260 proteins or 81% of them are annotated. We consider a biological process annotation informative if it has these two properties. First, at least *k* proteins are annotated with it. Second, each of its descendant’s GO terms needs to have less than *k* proteins annotated with them. We set 3 as a value of *k* and we note that 16,021 biological processes corresponding to these 25,260 proteins that are participating in our interactions. We also use 332 human proteins involved in 26 proteins of the SARS-CoV-2 virus that were revealed by Gorden et al. [14]. The set, *T*, shows these 332 proteins as possible targets of the SARS-CoV-2 virus. For this set of 332 human proteins, we also consider 1374 IBP GO terms as high-confidence SARS-CoV-2 Human PPI.

We define the overlap between two biological processes, $p_1$ and $p_2$ in the following way (| . | shows the size):

4 $$\begin{aligned} Overlap(p_1,p_2 )=\frac{|p_1\bigcap p_2|^2}{|p_1||p_2|}. \end{aligned}$$

Then, the processes with more than 15% overlaps have been removed. Through this filtering method, we have 1213 non-overlapping biological processes corresponding to COVID-19.

#### Interactive information between drugs and human protein targets

To evaluate our candidate targets, we use all drugs and their corresponding targets interactions that are reported in the UniProt [24]. These interactions contain 6163 drugs from All-Drug group that are reported in UniProt, these drugs have 2898 protein targets. We also use 44 experimental unapproved drugs for COVID-19 that are reported in DrugBank [26]. From these 44 drugs, 27 drugs have no target information and only 17 drugs have the drug target information. These 17 drugs can target 78 proteins in a cell. This group of drugs is denoted as Covid-Drug. The second group of drugs contains 590 drugs as clinical trials for COVID-19. From these 590 drugs, 328 drugs have targets in the PPI network denoted as Clinical-Drug. These 328 drugs can target 888 proteins in a cell.

#### Construction of drug–target network

We define some topological features in a PPI network for cluster identification of drugs. These features cluster the available experimental unapproved drugs for COVID-19 with respect to these topological properties of their associated targets in the PPI network. To do this, we define a drug–targets network in the following way.

Each drug–targets network is considered as a bipartite graph $H= <D, \tau , E^*>$. In graph *H*, nodes are divided into two different sets. The first one, *D*, demonstrates the set of experimental unapproved drugs for COVID-19, and the second one, $\tau$, shows the experimental unapproved drug targets. Each edge $e_{vd} \in E^*$ shows that two nodes $v \in \tau$ and $d \in D$ are connected if the node *v* in a human cell be a target of drug *d*. In fact $\tau$, contains the proteins that are placed in the intersection of all drug targets with 2898 proteins and set *E*. Supposed that $G=<V, E>$ is a PPI network that contains the set of virus targets (*T*) and the set of main targets ($\tau$). Two nodes $v_i$ and $v_j$ are neighbors if there is an edge between them. Suppose that $N(v_i)$ shows a set of all neighbors for a node $v_i$, therefore $d(v_i) = | N(v_i)|$ indicates the degree of $v_i$.

We define 3 different informative topological features for each drug, *d*, and its particular targets as follows. The following features are defined with respect to the situation of its main targets and COVID-19 related biological process.

1. $D_T (d)$: The average ratio of the number of neighbors for each protein $v_i \in \tau _{d}$ that is also placed in set *T* according to the degree of $v_i$.
  5 $$\begin{aligned} D_T (d) = \frac{\sum _{i=1}^{m} { \frac{|N(v_i) \bigcap T|}{ d(v_i)} }}{ |\tau _{d}|}, \end{aligned}$$
  where $\tau _{d} = \{ v_1, ....v_m \}$ denotes the number of main targets for drug *d*.
2. The participation rate of $\tau _{d}$ in set $\pi$ defines as follow:
  6 $$\begin{aligned} P_{IBP} (d) = 1- \sum _{p_i \in \pi } \left( { \frac{ | p_i \bigcap \tau _{d} | }{ \sum _{p_i \in \pi } {| p_i \bigcap \tau _{d} |} } } \right) ^2, \end{aligned}$$
  where set $\pi = \{p_1,p_2, ...,p_k \}$ shows the non-overlapping biological processes corresponding to COVID-19. The possible values for $P_{IBP} (d)$ is between 0 and 1. If the value of $P_{IBP} (d)$ is closer to 1, it means the neighbors of node *d* have higher distribution in the set of biological processes.
3. $D_P (d)$: The average ratio of the number of neighbors for each protein $v_i \in \tau _{d}$ that is also placed in set $\pi$ according to the degree of $v_i$.
  7 $$\begin{aligned} D_P (d) = \frac{\sum _{i=1}^{m} { \frac{|N(v_i) \bigcap P|}{ d(v_i)} }}{ |\tau _{d}|}. \end{aligned}$$
  where $$P=\bigcup _ {p_i \in \pi } p_i$$.

#### Clustering method based on topological features of drug targets

Suppose that $G=<V, E, \omega>$ is a COVID-19 related biological network and $H=< \tau , D, E^*>$ is a bipartite drug–target graph $(\tau \subset V)$. Let $E \subset V$ be a set of essential proteins and $\tau$ be a set of main targets. Now, for each drug that has at least one target in set $\tau$, we measure the topological features $D_P$, $D_T$ and $P_{IBP}$ with respect to their targets in $\tau$ . Suppose that $d^*$ is a drug from the Covid-Drug group with the corresponding values of topological features $D_P(d^*)$, $D_T (d^*)$ and $P_{IBP} (d^*)$, respectively. According to the near zero threshold $\epsilon$, a drug *d* is placed in the same cluster with $d^*$ if the following equation is satisfied:

8 $$\begin{aligned} C = \{ d \in D \ \ \ \& \ \ | \sqrt{ { {( D_P(d) - D_P(d^*)) ^2} + {(D_T (d) - D_T (d^*))^2} } + {( P_{IBP} (d) - P_{IBP} (d^*)) ^2} } | < \epsilon \}. \end{aligned}$$

**Fig. 1:**
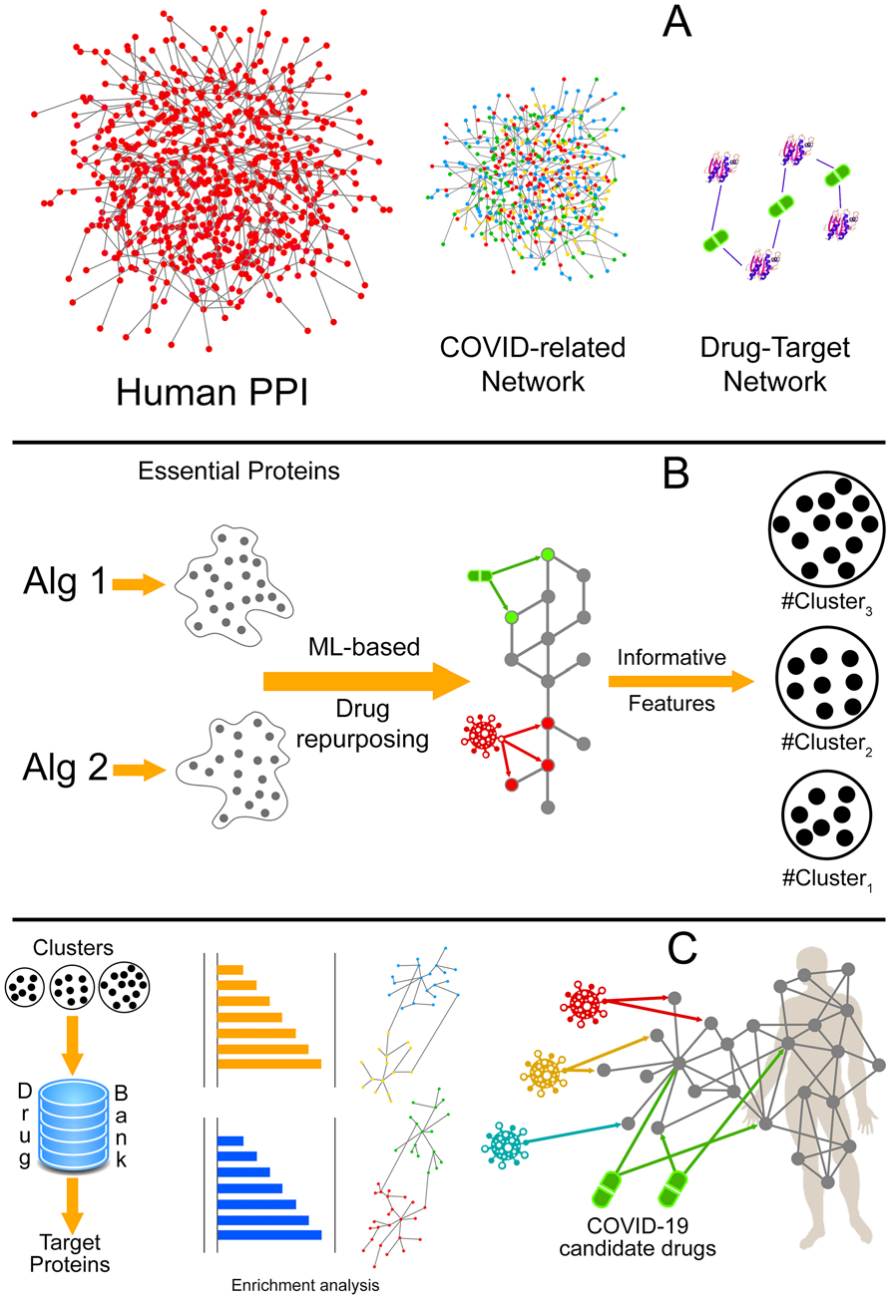
Overall workflow. Our method integrates a drug–target network with a Human-virus network in the human PPI network. **A**. Human and coronavirus host proteins were collected from different datasets to generate a COVID-19 related biological network. **B**. Algorithm 1 (Alg 1) and 2 (Alg 2) detect the essential proteins in the COVID-19 related biological network. Three Informative features introduced. The Machine Learning method used these features and find three significant clusters. **C**. The resulted clusters evaluated with different measures. These measures are based on drug targets in these clusters. Finally, some candidate drugs recommended

The overall view of our proposed method is illustrated in Fig. 1. Human and coronavirus host proteins were collected from different datasets to generate a COVID-19 related biological network (Part (A)). In Part B, Algorithm 1 (Alg 1) and 2 (Alg 2) are applied to detect the essential proteins in the COVID-19 related biological network. According to the defined features, the clustering method was used to find five appropriate clusters. In Part (C), the resulted clusters evaluated with different measures and some candidate drugs recommended.

## Results

### Evaluation essential proteins related to COVID-19

The protein sets that are resulted from algorithms 1 and 2 are placed in the sets $Cut_1$ and $Cut_2$, respectively. The union of $Cut_1$ and $Cut_2$ is placed in the $Cut_{union}$ set and the intersection of them is placed in $Cut_{intersect}$, respectively. For more evaluation of essential proteins of $Cut_1$ and $Cut_2$ sets, we study the topological properties of these two sets. In this work, we claim that through our cut sets as results of two presented algorithms, the maximum number of IBP GO terms are disrupted. We also claim that the two disjoint sets of vertices $G_1$ and $G_2$ (resulting from the cut set) are approximately equal in size. Moreover, each IBP GO term, like *C* has almost the same size on both sides of $G_1$ and $G_2$ sets. Suppose that *C* is a process from the IBP GO terms. The disruption score for this process is defined as follows [16]:

9 $$\begin{aligned} Score_{disrupt(C)} = \frac{MAX \{ C \cap G_{1}, C \cap G_{2} \}}{|C|}, \end{aligned}$$

The closer value of $Score_{disrupt(C)}$ to $\frac{1}{2}$ indicates that process *C* is completely disrupted. However, if the $Score_{disrupt(C)}$ for a process *C* is in the range $[0,\frac{1}{2}+ \epsilon ]$, we say that this process is $\epsilon$-disrupted.

For more evaluation of our proposed cut sets, $Cut_1$ and $Cut_2$, we define two other cut sets with some important topological features. We want to compare our proposed cut sets with these two cut sets and show the advantage of our proposed cut sets with respect to the defined measure $Score_{disrupt(C)}$. In the first cut set, we select the high degree vertices such that the removal of these vertices divides the graph *G* into two disjoint parts. This set contains hubs and we named this set as $Cut_{hub}$. In the second cut set, we select the high weight vertices such that the removal of these vertices divides the graph *G* into two disjoint parts. We named this set as $Cut_{weight}$.

**Table 1:** The number of $\epsilon$-disrupted processes for the selected cut sets

	$Cut_1$	$Cut_2$	$Cut_{hub}$	$Cut_{weight}$
0.05-disrupted	745	750	230	298
0.1-disrupted	877	892	378	403
0.2-disrupted	1021	1037	682	733

In Table 1 we compare the number of $\epsilon$-disrupted processes for $Cut_1$, $Cut_2$, $Cut_{hub}$ and $Cut_{weight}$ respectively. The results of Table 1 show that $Cut_1$ and $Cut_2$ have better disruption properties and this confirms that the selection algorithm that we used for $Cut_1$ and $Cut_2$ are significantly better than other algorithms.

In Table 2, we study the number of IBP GO terms that are disrupted through these cut sets. We find that almost all sets disrupt a high number of biological processes. However, $Cut_{union}$ shows significant superiority with respect to the number of drugs in Covid-Drug and Clinical-Drug groups. Table 2 shows that from these 17 drugs in the Covid-Drug group, 16 drugs are approved with $Cut_{union}$, and from 328 drugs in the second group as Clinical-Drug, 273 drugs are approved with $Cut_{union}$. Therefore, the set $Cut_{union}$ could be a possible good candidate to find essential proteins related to COVID-19 pathology as drug targets. To find these essential proteins, we use gene-disease relationship from DAVID tools. We also study some essential proteins in $Cut_{union}$ that are shared by cardiovascular-related, hypertension, diabetes type 2, kidney-related and lung-related diseases and placed them in a set *E*. Table 3 shows 93 proteins of set *E* that are annotated to four out of five of these specific diseases with significant p-values. We also find that from 17 drugs in the Covid-Drug group, 10 drugs including Bevacizumab, Azithromycin, Ritonavir, Ibuprofen, Colchicine, Darunavir, Methylprednisolone, Tocilizumab, Chloroquine, and Dexamethasone. The results also show that from 328 drugs in Clinical-Drug, 185 drugs are approved by set E. Generally, among 6,163 drugs from the All-Drug group, 1689 drugs are approved by set E. These drugs target 65 proteins out of 93 proteins in set E. We also evaluate set E with respect to the related pathways with DAVID tools. The results show significant pathways related to COVID-19 that have been studied by previous studies [27–30]. A part of these pathways with significant p-values are reported in Table 4.

**Table 2:** The first row shows the number of proteins in sets *T*, $\hbox {Cut}_{2}$, $\hbox {Cut}_{{1}}$, $\hbox {Cut}_{{intersect}}$ and $\hbox {Cut}_{{union}}$, respectively.

	*T*	$\hbox {Cut}_{2}$	$\hbox {Cut}_{{1}}$	$\hbox {Cut}_{{intersect}}$	$\hbox {Cut}_{{union}}$
No. Protein	332	2017	2100	1115	3002
IBP Go terms	1374	1279	1197	1120	1306
No. targets in Covid-Drug	1	22	20	15	27
No. targets in Clinical-Drug	15	218	217	154	281
No. approved drugs in Covid-Drug	2	15	15	14	16
No. approved drugs in Clinical-Drug	30	246	260	225	273

The number of IBP GO terms overlapped with these subsets collected in the second row. The number of drug targets in each drug group that are associated with these subsets are reported in the third, fourth and fifth rows, respectively. The number of drugs in each drug group that are associated with these subsets are reported in the sixth, seventh and eighth rows, respectively

**Table 3:** Essential protein related to COVID-19 pathology

Essential protein related to COVID-19 pathology	
O00206, O14543, O14763, O60603, P00533, P00734, P01019, P01033, P01130, P01133, P01137, P01344	
P01374, P01375, P01579, P01583, P01584, P01889, P01891, P01892, P01911, P01912, P02647, P02649	
P02751, P02778, P03372, P03989, P04114, P04229, P04439, P04637, P05019, P05089, P05106, P05112	
P05164, P05231, P05362, P05534, P06858, P08253, P08571, P08684, P09211, P09601, P10145, P10415	
P10635, P11021, P11226, P11473, P13498, P13500, P13501, P14210, P14780, P15692, P16035, P17813	
P19438, P19838, P21549, P25445, P28482, P29279, P29459, P29474, P31645, P31749, P35222, P35354	
P38936, P40763, P40933, P41597, P42336, P42345, P42898, P48023, P48061, P60568, P78423, P78527	
P81172, Q04721, Q14116, Q15848, Q16236, Q30201, Q99958, Q9NR96, Q9Y2R2

**Table 4:** Some of the significantly enriched pathways that are related to COVID-19 essential proteins (E)

Annotation cluster 1	Enrichment score: 11		
Term	Count	P value	
hsa05142:Chagas disease (American trypanosomiasis)	20	2.47E−18	[27]
hsa05323:Rheumatoid arthritis	17	1.79E−15	[27]
hsa05144:Malaria	14	4.85E−15	[27]
hsa05321:Inflammatory bowel disease (IBD)	15	7.61E−15	[27]
hsa05140:Leishmaniasis	14	8.80E−13	[27]
hsa05152:Tuberculosis	19	9.13E−13	[27]
hsa04620:Toll-like receptor signaling pathway	15	1.15E−11	[28]
hsa05146:Amoebiasis	14	1.75E−10	[27]
hsa05145:Toxoplasmosis	14	2.81E−10	[27]
hsa05134:Legionellosis	10	8.56E−09	[29]
hsa05133:Pertussis	10	1.66E−07	[27]
hsa04621:NOD-like receptor signaling pathway	9	2.12E−07	[30]

Annotation cluster 2	Enrichment score: 8.80		
Term	Count	P value	
hsa05168:Herpes simplex infection	20	1.31E−13	[29]
hsa04940:Type I diabetes mellitus	12	8.30E−13	[27]
hsa05332:Graft-versus-host disease	11	1.89E−12	
hsa05330:Allograft rejection	10	2.35E−10	[27]
hsa05320:Autoimmune thyroid disease	7	2.72E−05	[27]

Annotation cluster 3	Enrichment score: 4.53		
Term	Count	P value	
hsa04931:Insulin resistance	10	3.80E−06	
hsa04920:Adipocytokine signaling pathway	8	1.47E−05	[28]
hsa05221:Acute myeloid leukemia	6	4.50E−04	

### Statistical properties of clusters

As we mentioned earlier, our clustering method introduces 5 different clusters named as $\#Cluster_1$, $\#Cluster_2$, $\#Cluster_3$, $\#Cluster_4$, and $\#Cluster_5$ for 1689 drugs that are approved by proteins in set *E*. Our clustering method uses the defined topological features and 10 approved drugs in Covid-Drug that mentioned in the previous subsection, as clustering criteria. In order to evaluate $\#Cluster_1$, $\#Cluster_2$, $\#Cluster_3$, $\#Cluster_4$, and $\#Cluster_5$, we compare our clusters with randomly generated sets. Suppose that $N_i=(a_i,b_i)$ for $i = 1,. . ., 10^5$ denotes both the number of Covid-Drug and Clinical-Drug in *i*-th randomly generated sets groups of size *n* (n=2, 8, 10, 28 and 14). In $N_i$, $a_i$ and $b_i$ denote the number of Covid-Drug and Clinical-Drug in group of size *n*, respectively. For *n* with sizes of 2, 8 , 10, 28 and 14, we define N=(1,1), (1,2) (1,3), (1,1), and (6,8), respectively. Let $X = \{i | N_i> N \}$ for $i = 1,..., 10^5$, where *X* denotes the number of random sets that performed better than the proposed clusters [31]. The null hypothesis, $H_0$, is that our selected drug set of size *n* is not important. The alternative hypothesis, $H_1$, is that our selected drug set of size *n* is indeed important. We use exceeding value (EV) as $EV=\frac{|X|}{100000},$ where |*X*| denotes the size of *X*. If $EV<\alpha$ then, we reject $H_0$ ($\alpha$ is a threshold value that we consider to be 0.05). The values of EV for $\#Cluster_1$, $\#Cluster_2$, $\#Cluster_3$, $\#Cluster_4$, and $\#Cluster_5$ are reported in Table 5 (These values cause extremely significant results). The EV denotes the percentage of random clusters that perform better than our clusters out of $10^5$ random selections. We can conclude that these values are extremely significant and our selected clusters show a better performance than all of these random selections and significantly far from random selection. In Table 6 we also report some details about the proposed clusters. In this table, the number of Covid-Drug, Clinical-Drug, and drugs that are placed in All-Drug groups are reported, respectively.

**Table 5:** The exceeding values (EV) for $\#Cluster_1$ ,$\#Cluster_2$, $\#Cluster_3$ ,$\#Cluster_4$ and $\#Cluster_5$

	EV values
$\#Cluster_1$	1E−05
$\#Cluster_2$	0.00018
$\#Cluster_3$	0.00109
$\#Cluster_4$	0.0011
$\#Cluster_5$	0.0013

**Table 6:** The number of All-Drugs, Covid-Drug and Clinical-Drug for $\#Cluster_1$, $\#Cluster_2$, $\#Cluster_3$, $\#Cluster_4$ and $\#Cluster_5$

	$\#Cluster_1$	$\#Cluster_2$	$\#Cluster_3$	$\#Cluster_4$	$\#Cluster_5$
No. All-Drugs	2	8	10	28	14
No. Covid-Drug	1	1	1	1	6
No. Clinical-Drug	1	2	3	1	8

### Evaluation of clusters with respect to proteins as drug targets

**Fig. 2:**
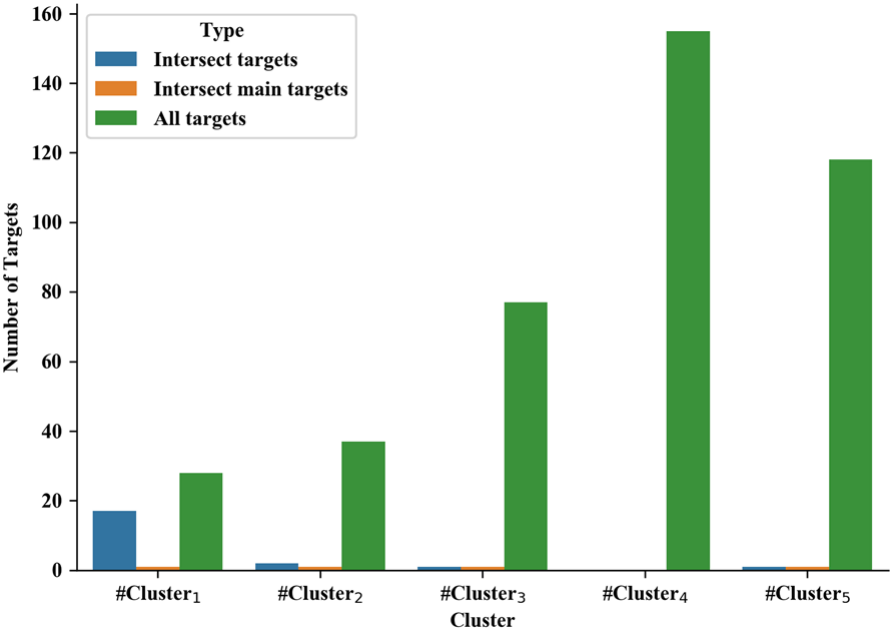
The blue columns show the common drug targets for each cluster. The orange columns show the number of main targets that are common between drugs in each cluster. The green columns show all of the targets for drugs in each cluster

For more evaluation, we investigate all of the proteins as drug targets in each cluster. Table 7 shows some details about our selected clusters. In this table, the first row indicates the number of proteins as drug targets in our PPI network. From these proteins, the number of important ones in each cluster that are mentioned as main targets is reported in the second row. These particular targets are the group of proteins from the first row of the table that is placed in set E. The number of human proteins that are targeted with the virus is demonstrated as a set *T* and reported in the third row. The fourth and fifth rows show the number of these proteins that are targeted through at least one drug in Covid-Drug and Clinical-Drug, respectively. Table 7 reports 10 drugs in $\#Cluster_3$ target 77 human proteins. From these 77 proteins, 10 proteins are reported as particular targets that are identified through our method. This cluster has the highest ratio of the main targets (10/77) in comparison with other clusters. Figure 2 illustrates the number of drug targets for each cluster. Drugs in each cluster can have distinctive and common targets. For example, the union of all targets for two drugs in $\#Cluster_1$ contains 28 proteins. From these 28 proteins, 17 of them are common between these two drugs. From these 17 proteins, 1 of them is placed in set *E* that is mentioned as main target.

**Table 7:** The first row indicates the number of proteins as drug targets in our PPI network

	$\#Cluster_1$	$\#Cluster_2$	$\#Cluster_3$	$\#Cluster_4$	$\#Cluster_5$
No. targets	28	37	77	155	118
No. main targets	2	2	10	10	14
No. targets in T	1	1	1	3	0
No. targets in Covid-Drug	23	18	29	43	47
No. targets in Clinical-Drug	28	32	69	140	107

From these proteins, the number of important ones in each cluster is reported in the second row. The number of human proteins that are targeted with the virus is reported in the third row. The fourth and fifth rows show the number of these proteins that are targeted through at least one drug in Covid-Drug and Clinical-Drug, respectively

Figure  2 also shows the number of main targets in each cluster. Each drug in these five clusters has one or multiple main targets. From these main targets three of them are common among all drugs of each clusters. These three main targets are Vascular Endothelial Growth Factor (VEGF)-A, Cytochrome P450 3A4 (CYP3A4), and Prostaglandin-endoperoxide synthase 2 (PTGS2) or Cyclo-oxygenase2 (COX-2), respectively. Despite the lack of evidence for COVID-19, previous research shows that the VEGF family (VEGFs) has a connection with COVID-19. A recent study shows that VEGFs are involved in “cytokine storm” inflammatory response. They claim that these genes may be used as prospective biomarkers for early diagnosis in COVID-19 patients [32]. The VEGFs can also be used for targeted drug delivery in COVID-19 treatment.

The second main target is PTGS2 or COX-2, which has been the subject of many studies on its association with COVID-19 and is a pro-inflammatory enzyme. Some studies showed that the structural proteins of the SARS-CoV family are reported to influence the expression of COX-2 and the increased expression of plasma PGE2 in the blood of SARS-CoV-infected patients. It is also reported that COX-2 plays a crucial role in limiting the anti-viral cytokine response to viral infection. Therefore, the use of an effective COX-2 inhibitor during early viral infection could enhance interferon responses. It might also increase anti-viral immunity [33]. The result of [34] study shows the importance of VEGF-A and COX-2 in relation to COVID-19. In this study, PPI analysis was used to find the hub genes linked to COVID-19 and lung cancer. Among the suggested hub genes, VEGF-A and COX-2 have been confirmed and could be used as biomarkers for COVID-19.

The next main target is Cytochrome P450 3A4 (CYP3A4). Cytochromes P450 (CYPs) is a superfamily of metabolizing enzymes. The CYP enzymes can be suppressed by an infection-related cytokine increase and inflammation. A recent study demonstrated that, like other viral infections, during the progression of COVID-19 local and systemic inflammation as well as the “cytokine storm” will potentially cause downregulation of the major CYP enzymes including CYP3A4 [35]. A new study proposed that COVID-19 pharmacogenetic studies include CYP3A4 variants [36]. The [33] study shows that CYPs metabolic activity will be surely changed during the SARS-CoV-2 infection in a similar manner, resulting in a pharmacokinetic interaction with the recommended drugs for COVID-19 treatments. In addition, liver involvement in COVID-19 may further complicate this problem, especially for drugs like remdesivir and chloroquine as COVID-19 treatments. Since remdesivir undergoes extensive metabolism by CYPs and chloroquine is also hepatically metabolized, understanding the nature of these drug-disease interactions is highly essential and can affect the therapeutic response of COVID-19 patients.

**Fig. 3:**
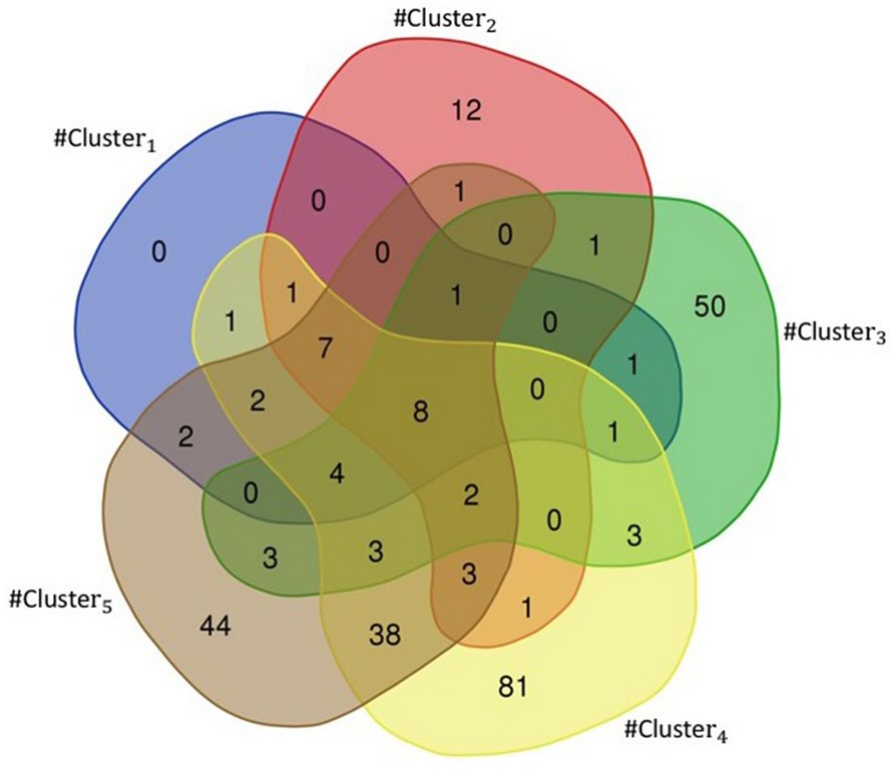
The Venn diagram shows the relation of targets for $\#Cluster_1$, $\#Cluster_2$, $\#Cluster_3$, $\#Cluster_4$ and $\#Cluster_5$

**Fig. 4:**
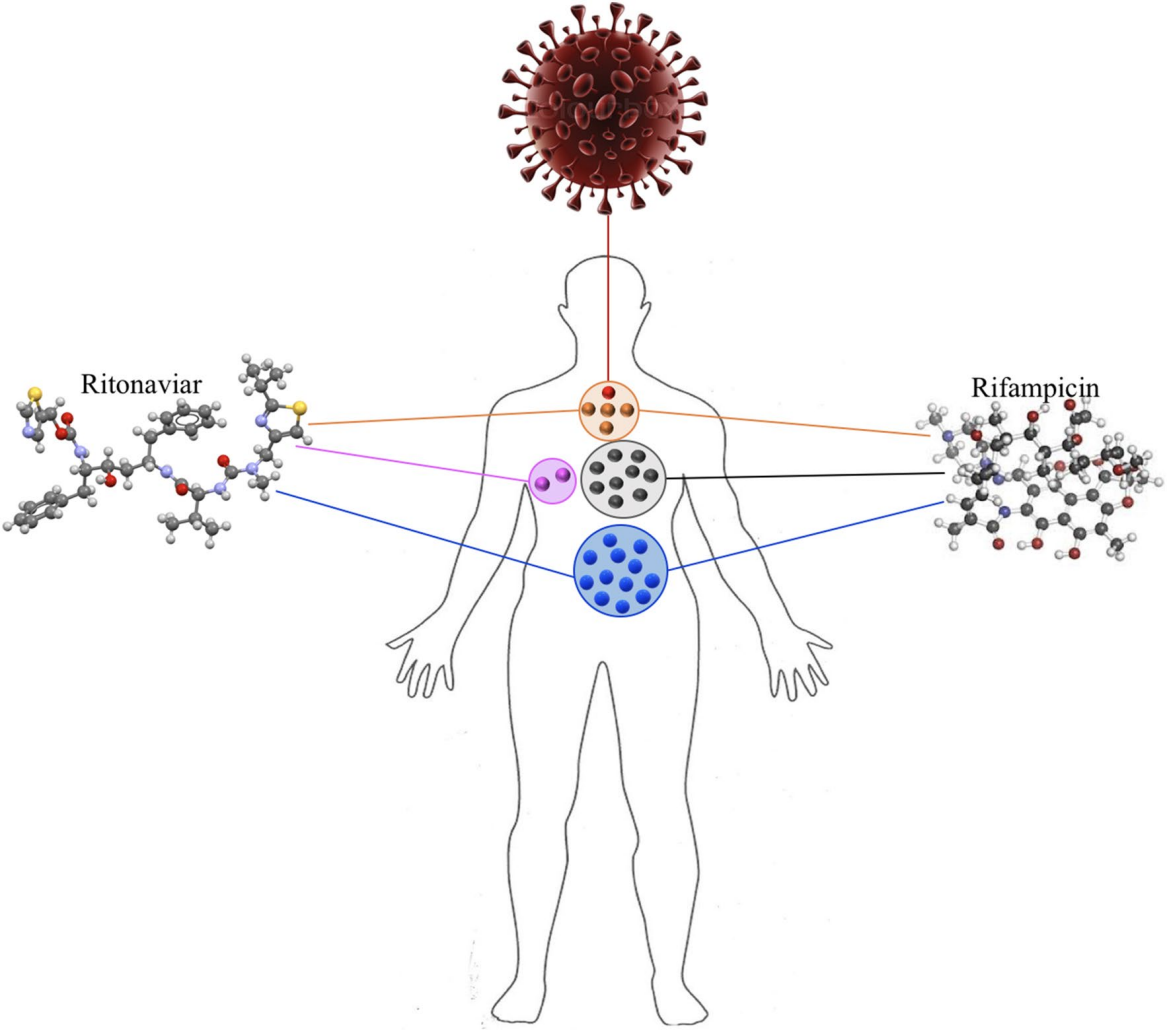
The example of common targets and total number of targets in $\#Cluster_1$

The Venn diagram in Fig. 3 illustrates the relationship between targets in 5 clusters. Our results show that despite the fact that the drugs in these five clusters are different from each other but they have 8 specific proteins as targets that are jointly targeted by the drugs in all five clusters. In addition, Fig. 3 shows that 64% (50/77) of the proteins as drug targets in $\#Cluster_3$ are different from the targets in other clusters, and all proteins as drug targets in $\#Cluster_1$ are targeted by at least one drug in the other clusters. In Fig. 4, as an example, we show that the common targets and the total number of targets that are selected through our method with respect to the defined topological features for $\#Cluster_1$.

**Fig. 5:**
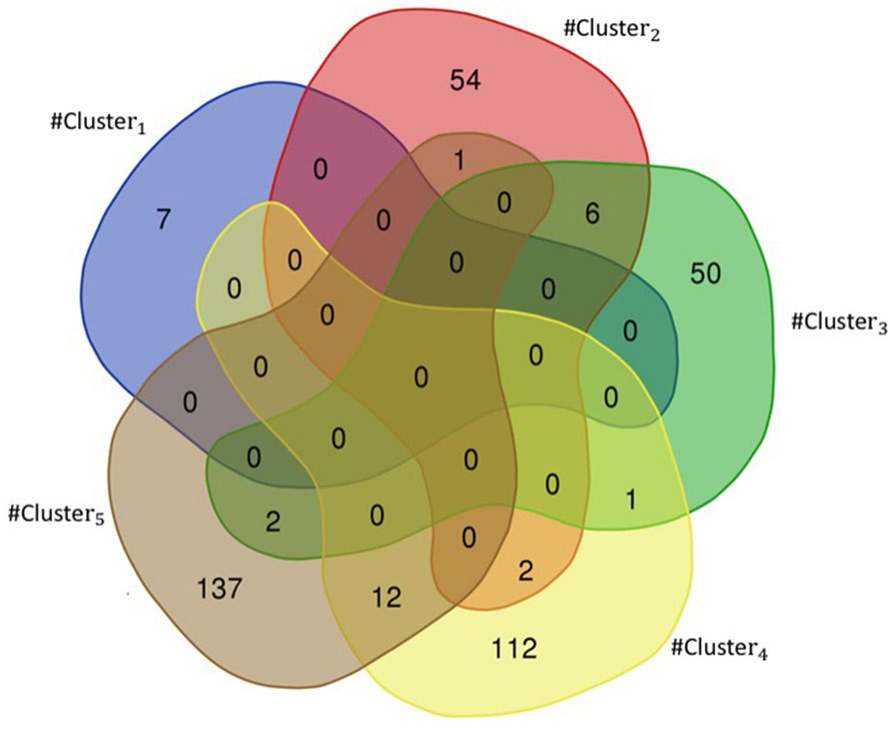
The relationship between diseases that are associated with drug targets in $\#Cluster_1$, $\#Cluster_2$, $\#Cluster_3$, $\#Cluster_4$ and $\#Cluster_5$

### Evaluation of clusters with respect to related diseases

We have studied the diseases associated with each of the drugs in each cluster based on the information on the Drugbank website. The Venn diagram in Fig. 5 shows the relationship between diseases that are associated with drug targets in each cluster. Figure 5 shows that there is no specific disease that is associated with all clusters. Respiratory Tract Infections and Type 2 Diabetes are two of six diseases that have common targets in$\#Cluster_2$ and $\#Cluster_3$. Diabetic Macular Edema (DME) is one of two diseases that have common targets in $\#Cluster_3$ and $\#Cluster_5$. Rheumatoid Arthritis and Ankylosing Spondylitis (AS) are two of twelve diseases that have common targets in $\#Cluster_4$ and $\#Cluster_5$.

### Drug repurposing candidate

In this section, the drug clusters identified with our proposed method have been analyzed to infer some useful drug repurposing candidates. Tables 8, 9, 10, 11 and 12 show repurposing candidates in $\#Cluster_1$, $\#Cluster_2$, $\#Cluster_3$, $\#Cluster_4$, and $\#Cluster_5$, contain 2, 8, 10, 28, and 14 drugs, respectively. Our clustering method enables us to partition the large drug-related network into smaller subgroups and this can simplify the drug repurposing process. Tables 8, 9, 10, 11 and 12 show the mechanism of action, therapeutic category, and supporting published evidence for each drug in $\#Cluster_1$, $\#Cluster_2$, $\#Cluster_3$, $\#Cluster_4$, and $\#Cluster_5$, respectively. From these 62 drugs, 10 of them have previously been proposed as potential drug repurposing candidates for COVID-19 disease.

**Table 8:** List of the repositioning candidates, therapeutic category and the supporting published evidence in $\#Cluster_1$

Candidate drug	Therapeutic category	References
*Ritonavir*	Anti-HIV agents and anti-infective agents	[26]
Rifampicin	Anti-bacterial agents and anti-infective agents	[38]

The drug in Clinical-Drug group is highlighted in italic

**Table 9:** List of the repositioning candidates, therapeutic category and the supporting published evidence in $\#Cluster_2$

Candidate drug	Therapeutic category	References
Mycophenolate mofetil	Anti-bacterial and anti-infective	[39]
Erythromycin	Anti-bacterial and anti-infective	[40]
*Azithromycin*	Anti-bacterial and anti-infective	[26]
Cerivastatin	Anticholesteremic agents	[41]
Ezetimibe	Anticholesteremic agents	[42]
Fusidic acid	Anti-bacterial and anti-infective	–
Canagliflozin	Alimentary tract and metabolism	[43]
Letermovir	Antiviral agents	[44]

Drugs in Clinical-Drug group are highlighted in italic

**Table 10:** List of the repositioning candidates, therapeutic category and the supporting published evidence in $\#Cluster_3$

Candidate drug	Therapeutic category	References
*Bevacizumab*	Angiogenesis inhibitors and antibodies	[26]
Minocycline	Anti-bacterial and anti-infective agents	[45]
Gliclazide	Alimentary tract and metabolism	[46]
Carvedilol	Adrenergic agents	[47]
Ranibizumab	Angiogenesis inhibitors and antibodies	–
Tromethamine	Drug delivery systems	[48]
Vandetanib	Antineoplastic agents	–
Veglin	–	–
Denibulin	Heterocyclic compounds	[49]
Foreskin keratinocyte	Allogeneic cultured cell scaffold	[50]

Drugs in Clinical-Drug group are highlighted in italic

**Table 11:** List of the repositioning candidates, therapeutic category and the supporting published evidence in $\#Cluster_4$

Candidate drug	Therapeutic category	References
* Aldesleukin*	Anti-infective agents	[26]
Dapsone	Anti-infective agents	[51]
Acetaminophen	Central nervous system agents	[52]
Celecoxib	Anti-inflammatory agents	[33]
Rofecoxib	Anti-inflammatory agents	[53]
Valdecoxib	Anti-inflammatory agents	[54]
Diclofenac	Anti-inflammatory agents	[55]
Triamcinolone	Alimentary tract and metabolism	-
Etoposide	Antineoplastic agents	[56]
Phenylbutazone	Anti-inflammatory agents	[57]
Meloxicam	Anti-inflammatory agents	[58]
Chlorphenesin	Central nervous system agents	[59]
* Ibuprofen*	Anti-inflammatory agents	[26]
Paclitaxel	Antineoplastic agents	-
Drospirenone	Contraceptive agents, female	[60]
Antipyrine	Anti-inflammatory agents	-
Etoricoxib	Anti-inflammatory agents	[53]
Resveratrol	Anti-inflammatory agents	[61]
Nimesulide	Anti-inflammatory agents	[62]
Capsaicin	Sensory system agents	[63]
Parecoxib	Anti-inflammatory agents	[64]
Pomalidomide	Antineoplastic agents	[65]
Cannabidiol	Antidepressive agents	[66]
Loxoprofen	Anti-inflammatory agents	[67]
Dexibuprofen	Anti-inflammatory agents	[68]
Propacetamol	Sensory system agents	[69]
Venetoclax	Antineoplastic agents	–
Nabiximols	Central nervous system agents	–

Drugs in Clinical-Drug group are highlighted in italic

**Table 12:** List of the repositioning candidates, therapeutic category and the supporting published evidence in $\#Cluster_5$

Candidate drug	Therapeutic category	References
*Colchicine*	Immunosuppressive agents	[26]
* Darunavir*	Anti-infective agents	[26]
* Dexamethasone*	Anti-inflammatory agents	[26]
*Methylprednisolone*	Anti-inflammatory agents	[26]
*Tocilizumab*	Antirheumatic agents	[26]
Acetaminophen	Central nervous system agents	-
* Chloroquine*	Anti-inflammatory agents	[26]
Donepezil	Central nervous system agents	[70]
Clomipramine	Central nervous system agents	[71]
Resveratrol	Anti-inflammatory agents	[72]
Curcumin	Anti-inflammatory agents	[73]
Curcumin sulfate	anti-inflammatory agent	[74]
Baricitinib	Antineoplastic and immunomodulating agents	[37]
Lidocaine	Antiarrhythmic agents	[75]

Drugs in Clinical-Drug group are highlighted in italic

## Discussion and summary

Researchers have been searching for efficient medications to prevent or cure COVID-19 since the first case was discovered in 2019. To advance this goal, we introduced the four steps method. In the first step, the COVID-19 related biological network was constructed and the essential proteins that have a wide range of important functions in the biological network were detected. In the second step, we focused on finding the most effective essential proteins related to COVID-19. To do this, we used two different algorithms to identify the minimum number of proteins that participate in a large number of IBP GO terms and placed them in two distinct sets. Then, we evaluated proteins of these two sets with respect to the number of approved Covid-Drug and Clinical-Drug by them (Table 2). We placed the union of these two sets in the set $Cut_{union}$ and studied set $Cut_{union}$ with respect to the number of IBP GO terms that are disrupted. As a result, the selected proteins can be identified as a suitable candidate set for the COVID-19. It is noticeable that not every essential protein is an appropriate candidate as an essential protein for COVID-19 pathology. Some of these essential proteins are related to the cellular function of the cell and selecting them as drug targets may lead to disruption of cellular function. Considering that, in the third step, we picked candidate proteins directly related to COVID-19 pathology. For the final essential protein selection process in this step, we identified proteins that were associated with underlying diseases such as cardiovascular disease, diabetes, hepatitis, lung, kidney diseases, and various types of cancer. Among 3,002 essential proteins related to COVID-19 in $Cut_{union}$, we detected 93 proteins associated with at least four of five underlying mentioned diseases as essential proteins related to COVID-19 pathology (Table 3). We evaluated these proteins with respect to the related pathways with DAVID tools (Table 4). As a result, these selected proteins could be suitable candidates as drug targets for COVID-19 treatment. In the fourth step, multiple informative topological features for drug–target and a PPI network were proposed. Our methods tried to find significant clusters containing appropriate candidate drugs through these features. These features cluster the available experimental unapproved drugs for COVID-19 into five groups ($\#Cluster_1$, $\#Cluster_2$, $\#Cluster_3$, $\#Cluster_4$, and $\#Cluster_5$). These clusters have a significant difference from random clusters (Table 5) and contain a significant number of Covid-Drug and Clinical-Drug (Table 6). We also used three different measures for validating the obtained clusters. The first measure was based on the proteins as drug targets in these clusters, we showed that the proposed clusters have meaningful targets that were known in recent studies as COVID-19 targets (Table 6 and Figs. 2, 3, and 4). The second measure was based on the related diseases that have drugs in our clusters. We found some related diseases like DME and Rheumatoid Arthritis that have drugs in two of our clusters (Fig. 5). The third measure was related to drugs as good candidates for drug repurposing in COVID-19 treatment.

In summary, the main advantage of our method in comparison to other studies was clustering FDA-approved drugs that are related to COVID-19 according to the biological and topological properties of their targets. It can be concluded that partitioning the drug-related network into smaller networks (clusters) can improve drug repurposing results for clinical trials. In this work, we proposed some good drug candidates as repurposing candidates for COVID-19 treatment. Our results showed that most of our drug candidates were used in clinical trials or suggested in at least one study as suitable drug repurposing candidates (Tables 7-11). Our results also revealed that the proposed informative features recommended some suitable candidate drugs like [37] and Rifampicin [38]. Finally, this study offered powerful network-based informative features for the fast identification of repurposable drugs as a potential treatment for COVID-19. The proposed method can effectively minimize the timing gap between preclinical testing conclusions and clinical results, which is a considerable problem in the fast development of efficient treatment strategies for the emerging COVID-19 outbreak.

## Data Availability

Datasets and the codes of the algorithms are available in our github repository, [https://github.com/rosaaghdam/Drug-Target.]

## Abbreviations

COVID-19: The coronavirus disease 2019
PPI: Protein−protein interaction
IBP: Informative biological processes
DAVID: Database for annotation, visualization, and integrated discovery
BioGRID: Biological general repository for interaction datasets
HIPPIE: Human integrated protein−protein inter-action reference
APID: Agile protein interactomes dataanalyzer
Hint: Homologous interactions
Y2H: yeast-two hybrid
UniProt: Universal protein resource
GO: Gene ontology
EV: Exceeding value
CYP3A4: Cytochrome P450 3A4
VEGF: Vascular endothelial growth factor
CYP3A4: P450 3A4
PTGS2: Prostaglandin-endoperoxide synthase 2
COX- 2: Cyclo-oxygenase2
CYPs: Cytochromes P450
